# Zero-fluoroscopy Radiofrequency Redo Ablation of Atrial Tachycardia Following Pulmonary Vein Isolation: A Tale of Two Systems

**DOI:** 10.19102/icrm.2018.091101

**Published:** 2018-11-15

**Authors:** Robert L. Percell, Erin D. Sharpe, Teresa M. Lassen, Steffani R. Maas, Lori J. Heiss, Dale A. Hansen

**Affiliations:** ^1^Electrophysiology Department, Bryan Heart Institute, Lincoln, NE, USA

**Keywords:** Atrial fibrillation, atrial tachycardia, catheter ablation, electroanatomic mapping, zero radiation

## Abstract

Regular atrial tachycardia (AT) is one of the most important proarrhythmic complications that may occur following left atrial pulmonary vein isolation (PVI). These tachycardias that develop after atrial fibrillation ablation may lead to worse symptoms than those from the original arrhythmia existing prior to the index ablation procedure. Ablation of various types of supraventricular tachycardias without the use of fluoroscopy has been shown to be feasible in both children and adults using three-dimensional mapping systems. We describe the case of a 71-year-old woman who developed a focal AT after a successful PVI procedure. The initial ablation failed with one mapping system. Repeat electrophysiologic study despite antiarrhythmic medications revealed the same focal AT, which was successfully ablated with a different mapping system. Both ablations were performed without fluoroscopy.

## Introduction

Regular atrial tachycardia (AT) or atrial flutter is one of the most important proarrhythmic complications that can occur following left atrial (LA) pulmonary vein isolation (PVI).^1^ The reported incidence of late ATs ranges from between 1% and 2.9% to between 10% and 24%.^2,3^ More extensive LA ablation, especially mitral lines and roof lines, are associated with a higher incidence of AT.^4^ Additionally, persistent atrial fibrillation (AF), increased LA size, and fibrosis may lead to increased AT development. Regular or organized ATs may occur weeks to months after the initial ablation.^5^ These tachycardias that develop after AF ablation may result in worse symptoms than those associated with the original arrhythmia prior to the index ablation procedure. Frequently, post-PVI ATs are often incessant and associated with rapid ventricular response.^6,7^ The mechanism of the AT is usually one of the following: focal, microreentrant, or macroreentrant.^8^ Frequently, conservative therapies, including antiarrhythmic medications, are not effective. Radiofrequency ablation procedures are mostly successful, but can be challenging, and require a complex approach.^9^

Ablation of various types of supraventricular and ventricular tachycardias without the use of fluoroscopy has been shown to be feasible in both children and adults, especially pregnant women, using nonfluoroscopic three-dimensional mapping systems (ie, zero-fluoroscopy approach). Also, it is feasible to use complex procedures such as PVI to treat AF without the use of fluoroscopy or protective lead aprons.^10^ Despite recent advances in electroanatomic mapping systems, the majority of cardiac electrophysiologists still rely on fluoroscopic techniques.^11^ There is no amount of radiation exposure that is known to be completely safe.^12^ Over time, both the stochastic and deterministic effects of radiation may lead to life-threatening cancers; therefore, the use of ionizing radiation should be as low as reasonably achievable (ALARA).^13^ Finally, a whole host of orthopedic issues may develop from the long-term use of protective lead aprons.^14^

## Case presentation

**Figure 1:**
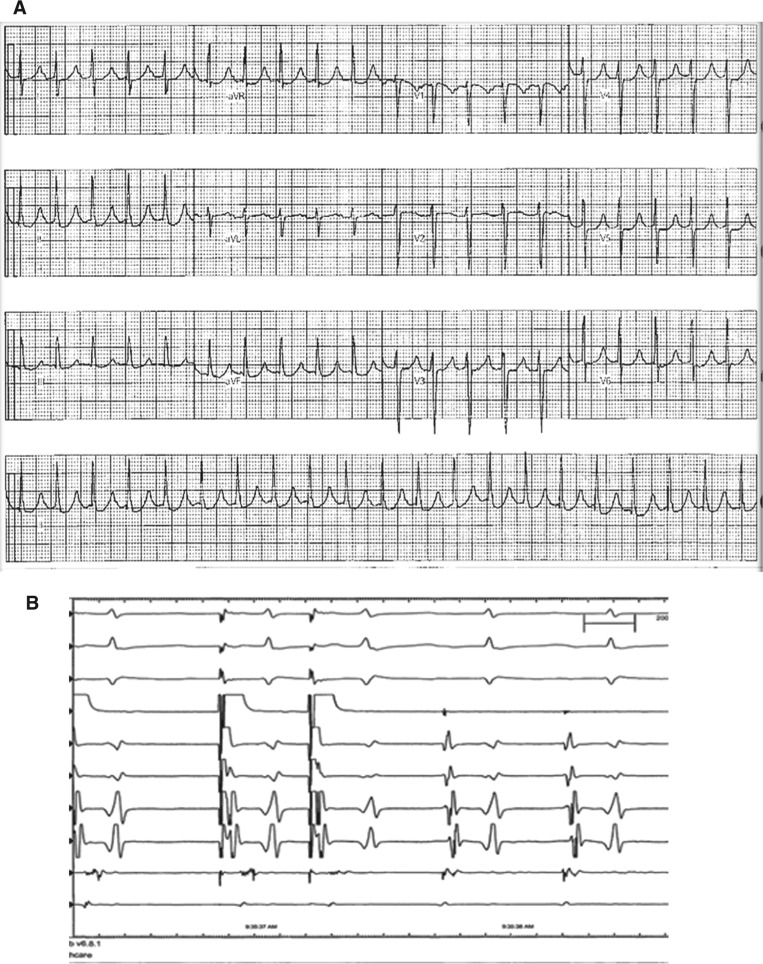
**A:** A 12-lead electrocardiogram revealing narrow-complex mid-RP tachycardia with rates of 470 ms. **B:** Intracardiac electrograms revealing CS9–10 pacing-induced tachycardia, with earliest activation at CS7–8.

**Figure 2:**
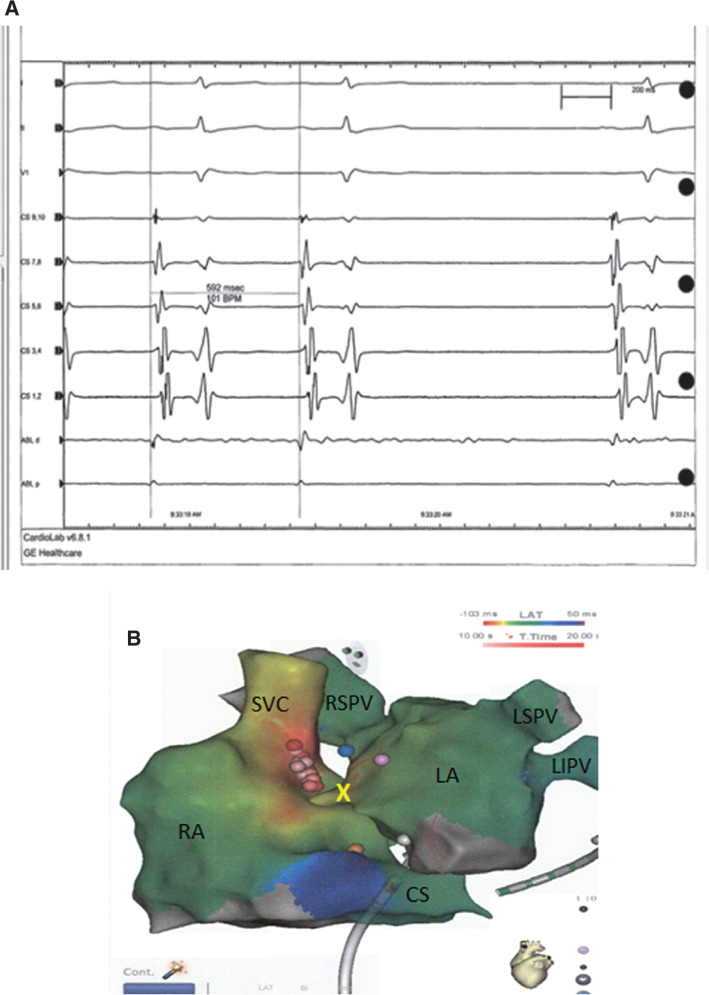
**A:** Intracardiac electrogram revealing slowing and termination of the tachycardia with ablation of the RSPV. **B:** CARTO^®^ 3 (Biosense Webster, Diamond Bar, CA, USA) map of the RA and LA in an anteroposterior top-down position. In the LA, the blue tag represents an ablation point adjacent to the RSPV with termination. In the RA, multiple ablation tags (pink and red) represent ablation points in the posteroseptal area and termination with the red tag. The yellow tag represents the His bundle, whereas the yellow X denotes the transseptal area. RA: right atrium; SVC: superior vena cava; RSPV: right superior pulmonary vein; LSPV: left superior pulmonary vein; LA: left atrium; LIPV: left inferior pulmonary vein; CS: coronary sinus.

After unsuccessful treatment with antiarrhythmic medications, a 71-year-old female was referred for treatment of drug-refractory, symptomatic, paroxysmal AF. She underwent an uncomplicated zero-fluoroscopy PVI procedure using a previously described technique without linear lesions.^10^ After confirming exit block of the pulmonary veins, she developed an intermittent regular, narrow-complex, mid- to long-RP tachycardia **(Figure 1A)** with a cycle length of 470 ms. The earliest activation in coronary sinus (CS) 7–8 was easily inducible with rapid atrial pacing **(Figure 1B)**. We were unable to entrain successfully, as the tachycardia would terminate. Based on these findings, we felt that this was most consistent with an AT. Using the CARTO^®^ 3 system (Biosense Webster, Diamond Bar, CA, USA) and mapping with the PentaRay^®^ multipolar catheter (Biosense Webster, Diamond Bar, CA, USA), the earliest activation appeared to originate outside of the right superior pulmonary vein (RSPV). The tachycardia appeared to terminate after a number of ablations at the ostium of the RSPV. The tachycardia was again reinitiated with atrial pacing **(Figure 2A)** with a similar cycle length and CS activation pattern. At this point, we mapped the right atrium (RA) and the earliest points were found to originate from the superoposterior portion of the RA near the superior vena cava junction **(Figure 2B)**. The distal ablation point was measured 10 ms early and the tachycardia ultimately terminated, though a number of ablation attempts were required **(Figure 2B)**.

**Figure 3:**
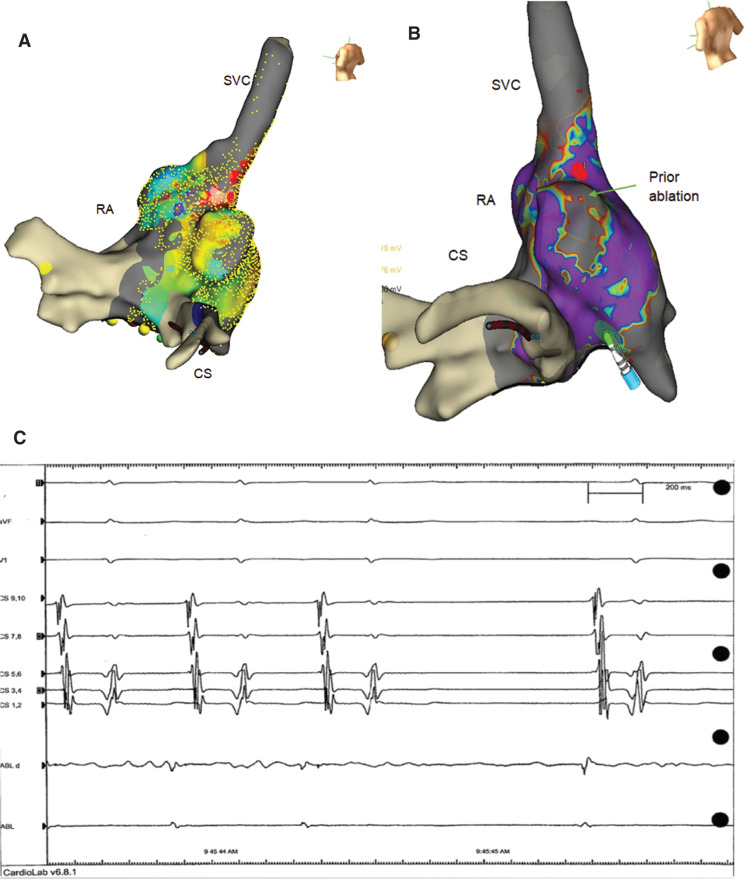
**A:** A three-dimensional EnSite Precision™ (Abbott Laboratories, Chicago, IL, USA) map of the RA in a posterior view revealing a high density of points. **B:** A three-dimensional EnSite Precision™ (Abbott Laboratories, Chicago, IL, USA) map of the RA in a posterior view revealing low-voltage (gray) areas at the site of prior ablation. The lone red spot denotes the single 15-second ablation attempt. **C:** Intracardiac electrogram revealing termination of the AT with restoration of sinus rhythm.

Unfortunately, the patient reported continued daily palpitations at four weeks after the procedure despite the use of medical therapy including ß-blockers, calcium channel blockers, and flecainide. Holter monitoring revealed what appeared to be the same AT with a similar cycle length. Repeat electrophysiologic study was performed with the EnSite Precision™ cardiac mapping system (Abbott Laboratories, Chicago, IL, USA), again without fluoroscopy. An alternative mapping system was used to obtain a higher-definition map with a greater number and concentration of points. AT was induced easily and, again, the superoposterior RA was affected earliest, with the same CS activation pattern and cycle length as noted previously. LA mapping with the Advisor™ FL Circular Mapping Catheter, Sensor Enabled™ (Abbott Laboratories, Chicago, IL, USA) revealed exit block in all four pulmonary veins without electrical activity. The entire cycle length was obtained with windows set from −97 ms to −99 ms. RA mapping was completed with a fill threshold of 20 units, with more than 7,500 points obtained in less than five minutes **(Figure 3A)**. Both the previous ablation site and the origin were easily located. A propagation map using local activation time clearly identified the “pebble in the pond” that seemed consistent with a focal AT **(Video 1)**. Utilization of the SparkleMap feature allowed for further visualization of the focality of this rhythm **(Video 2)**. Ablation at a site 20 ms early and adjacent to the prior ablation terminated the tachycardia with one burn **(Figure 3B)** at 15 seconds **(Figure 3C)**. The tachycardia was no longer inducible with burst pacing, even in the presence of isoproterenol. The patient was discharged the following day without complications. She has not had a recurrence in the six months off flecainide to date and is maintained solely on low-dose ß-blocker therapy for hypertension.

## Discussion

This case demonstrates a number of pertinent points. First, post-PVI AT is common and can be very symptomatic for patients. Additionally, ATs may be more difficult to treat/cure than the original paroxysmal AF for which the index ablation procedure was performed. The origin of the AT may not be in the original chamber first identified, as was true in this case wherein the AT originated from the RA and not the RSPV. The proximity of the RSPV and the RA including interatrial connections are well described. Furthermore, there are differences among the different mapping systems: while some use magnetic data solely (eg, CARTO^®^ from Biosense Webster, Diamond Bar, CA, USA), others use both magnetic and impedance data for mapping (eg, EnSite Precision™ from Abbott Laboratories, Chicago, IL, USA) and are reported to have up to 27 times greater capability in point acquisition density.^15^ To add to this, both types of systems can deliver propagation maps with local activation time. However, only the latter (ie, EnSite Precision™ from Abbott Laboratories, Chicago, IL, USA) has the SparkleMap feature that allows the operator to view live peak-to-peak voltage activation. More recently, however, CARTO^®^ (Biosense Webster, Diamond Bar, CA, USA) has added the Ripple Mapping feature, which has similar capabilities. Finally and most importantly, the complex ablation of post-PVI AT, including even redo procedures using two different mapping systems, may be completed safely without using harmful radiation. All electrophysiologists performing ablations with fluoroscopic radiation should meticulously scrutinize fluoroless techniques in order to reduce the amount of ionizing radiation to themselves, their patients, and staff and to eliminate mechanical disorders from long-term use of protective lead aprons.

## Conclusion

Complex ablation of post-PVI AT may be performed safely using a zero-fluoroscopy approach with multiple high-density mapping systems, which may improve outcomes in redo ablation cases.
